# A new modified MR dual precision positioning of thin-slice oblique sagittal fat suppression proton density weighted imaging: its diagnostic accuracy in anterior cruciate ligament injury

**DOI:** 10.1038/s41598-023-50909-4

**Published:** 2024-01-03

**Authors:** Kun Li, Felix Young Jhonatan, Zhaohui Yu, Jiachen Liu, Lixin Huang, Huilin Yang, Jun Du

**Affiliations:** 1 Department of Orthopedic Surgery, The First Affiliated Hospital of Soochow University, No. 899 Pinghai Road, Suzhou, 215000 China; 2 Department of Orthopedic Surgery, The Affiliated Wuxi People’s Hospital of Nanjing Medical University, No. 299 Qingyang Road, Wuxi, 214023 China; 3 Department of Orthopedic Magnetic Resonance Chamber, The First Affiliated Hospital of Soochow University, No. 899 Pinghai Road, Suzhou, 215000 China

**Keywords:** Trauma, Diagnosis, Magnetic resonance imaging

## Abstract

To evaluate the diagnostic accuracy of a new modified MR dual precision positioning of thin-slice oblique sagittal fat suppression proton density-weighted imaging (DPP-TSO-Sag-FS-PDWI) sequence in detecting ACL injuries and its grades compared to standard sequences using arthroscopy as the standard reference. 42 patients enrolled in this retrospective study received the 1.5-T MRI with standard sequences and the new modified DPP-TSO-Sag-FS-PDWI sequence, and their arthroscopy results was recorded. The Mc Nemer-Bowker and weighted Kappa was performed to compare the consistency of MRI diagnosis with arthroscopic results. Finally, the diagnostic accuracy was calculated based on the true positive, true negative, false negative and false positive values. The diagnostic consistency of the DPP-TSO-Sag-FS-PDWI were higher than standard sequences for both reader 1 (*K* = 0.876 vs. 0.620) and reader 2 (*K* = 0.833 vs. 0.683) with good diagnostic repeatability (*K* = 0.794 vs. 0.598). Furthermore, the DPP-TSO-Sag-FS-PDWI can classify and diagnose three grades of ACL injury [the sensitivity, specificity, accuracy, positive predictive value (PPV) and negative predictive value were more than 84%], especially for grade II injury as the PPV was superior for reader 1 (92.3% vs. 53.9%) and reader 2 (84.6% vs. 69.2%). The new modified DPP-TSO-Sag-FS-PDWI sequence can display the ACL injury on one or continuous levels by maximizing the acquisition of complete ligament shape and true anatomical images, and excluding the influence of anatomical differences between individuals. It can improve the diagnostic accuracy with good repeatability and classify three grades of the ACL injury.

## Introduction

Anterior cruciate ligament (ACL) arises from the medial surface of the lateral femoral condyle and extends the oblique course to the medial and anterior portion in the central area of the tibial plateau^1^. The ligament is divided into two bundles, including the anteromedial bundle and the posterolateral bundle. The function of ACL is to maintain anterior tibial movement and prevent excessive tibial rotation^2^. The ACL injury is the most common ligament injury in the United States, with 100,000 to 200,000 yearly^3^. Internal or external rotation and valgus or varus movements increase the risk of ACL damage^3,4^. Meanwhile, a majority of ACL injuries occur when the knee is fully stretched and sudden fall^2,3^. A complete tear of ACL mainly occurs in femoral insertion due to anterior tibial displacement and increased internal rotation, resulting in instability of the knee joint and a significant increase in rates of damage to menisci or articular cartilage^3,5^.

The examination under anesthesia and visual investigation at the knee arthroscopy are the gold standard by observing the internal structure of the joint, but most patients undergo magnetic resonance imaging (MRI) before arthroscopic treatment^6^. MRI is an effective method for the diagnosis of knee injury because it’s non-invasive and lacks of ionizing radiation to the patients^7,8^. It also minimized the opportunity for unnecessary arthroscopic surgery, which required high financial needs and possibly trauma^9^. MRI displayed the injured site with excellent soft-tissue contrast and high-resolution image with wide range parameter and direct observation of ACL morphologic characteristics^8–10^. However, the limitation of standard MRI sequences [including sagittal and coronal T_1_-weighted imaging (T_1_WI), T_2_WI and fat suppression proton density-weighted imaging (FS-PDWI)] is that it produces partial volume effect caused by relatively slice thickness (3–5 mm) and increased gap (10–30% of the thickness) between slices^11,12^, which is a difficult challenge to diagnose partial tear and the diagnostic accuracy was relatively low^13,14^.

A sagittal-oblique technique has been proposed to standard sequences for the detection of partial ACL tears. However, these sequences have not been assessed for diagnostic accuracy based on arthroscopy as a standard^15^. Therefore, we added a new modified dual precision positioning of thin-slice oblique sagittal fat suppression proton density-weighted imaging (DPP-TSO-Sag-FS-PDWI) to standard sequences to improve the diagnostic accuracy by enhancing the visualization of ACL natural anatomical structure (scanning along the ACL anatomical direction on both axial and coronal planes), and eliminating partial volume effects (reducing the slice thickness from 5 to 2.3 mm and thickness gap from 0.5 to 0 mm). The purpose of this study was to evaluate the diagnostic accuracy of a new modified DPP-TSO-Sag-FS-PDWI sequence in detecting ACL injuries and its different grades compared to standard sequences using arthroscopy as the standard reference.

## Material and methods

### Study population

This retrospective study was conducted according to the guidelines of the Declaration of Helsinki, and approved by the Institutional Ethics Committee of the First Affiliated Hospital of Soochow University (protocol code No.155, 13th of July 2020). Informed consent was waived by the Institutional Ethics Committee of our hospital due to the retrospective nature of the study and the use of anonymous clinical data. We searched the data of patients from January 1, 2017, to January 1, 2020, who received MRI and arthroscopy examination of the knee joint. We excluded patients without the new modified DPP-TSO-Sag-FS-PDWI sequence, the scanning procedure is performed in other institutions and without arthroscopic examination. Finally, forty-two patients were included in the study, all of whom performed MRI examination with the new modified DPP-TSO-Sag-FS-PDWI sequence and recorded arthroscopic examination during surgery (Table 1).

**Table 1:** Clinical data of the participants with MRI examination and arthroscopy results.

Sex	Age	Right knee	Left knee	Grade of injury	0	I	II	III
33 men	29 ± 7 (19–54)	20	13	Arthroscopy	2	3	13	24
9 women	31 ± 12 (14–52)	3	6	Standard sequence 1	3	4	8	27
				Standard sequence 2	3	5	12	22
				DPP-TSO-Sag-FS-PDWI 1	2	3	14	23
				DPP-TSO-Sag-FS-PDWI 2	2	3	13	24

DPP-TSO-Sag-FS-PDWI, Dual precision positioning of thin-slice oblique sagittal fat suppression proton density weighted imaging; 1, Reader 1; 2, Reader 2.

### Image acquisition

**Figure 1:**
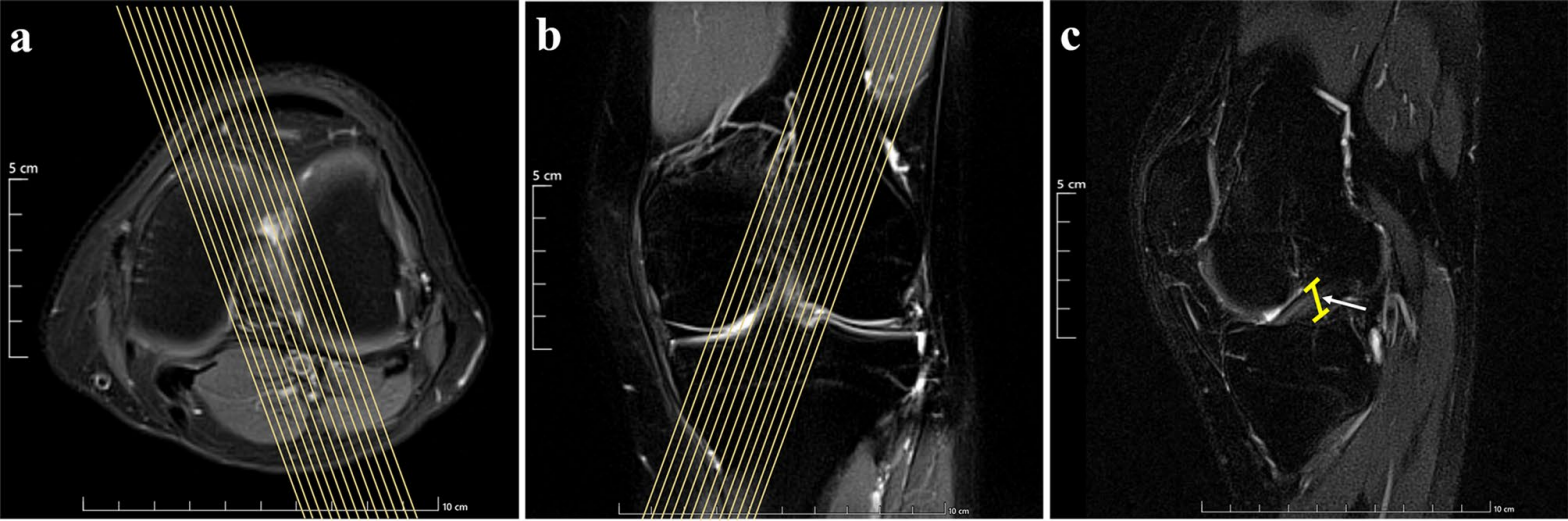
Scanning method for the new modified DPP-TSO-SAG-FS-PDWI sequence. The left knee of a 26-year-old man was displayed on (**a**–**c**). ACL on the new modified DPP-TSO-Sag-FS-PDWI sequence (**c**) were performed along its anatomic direction on axial and coronal PDWI (as yellow line marked on (**a** and **b**)). Its complete structure was shown in the yellow range (arrow on (**c**)) with low signal intensity.

MRI scanning was performed on a 1.5-T superconducting magnetic resonance scanner (Magnetom Espree, A Tim System; Siemens, Erlangen, Germany) with a single channel knee coil. The patients were scanned in the supine position and their legs were straightened. The knee joint was fixed by sandbags on both sides and the lower edge of the patella was placed in the center of the coil. The scanning range was initiated from the distal end of the femur to the proximal end of the tibia and fibula, including the entire knee joint. The conventional MR scanning sequences and scanning order are axial fat suppression proton density-weighted imaging (FS-PDWI) [repetition time/echo time (TR/TE), 2970 ms/36 ms; field of view (FOV), 180 mm; matrix size, 256 × 256; slice thickness/slice spacing, 4.0 mm/1.2 mm], sagittal T_1_WI (TR/TE, 350 ms/11 ms; FOV, 160 mm; matrix size, 320 × 320; slice thickness/slice spacing, 4.0 mm/0.6 mm), sagittal FS PDWI (TR/TE, 2340 ms/43 ms; FOV, 160 mm; matrix size, 256 × 256; slice thickness/slice spacing, 4.0 mm/0.6 mm), coronal T_2_WI (TR/TE, 3052 ms/63 ms; FOV, 160 mm; matrix size, 320 × 320; slice thickness/slice spacing, 4.0 mm/0.6 mm) and coronal FS PDWI (TR/TE, 2340 ms/43 ms; FOV, 160 mm; matrix size, 256 × 256; slice thickness/slice spacing, 4.0 mm/0.6 mm). Finally, the DPP-TSO-Sag-FS-PDWI sequence (TR/TE, 2000 ms/42 ms; FOV, 160 mm; matrix size, 320 × 320; slice thickness/slice spacing, 2.3 mm/0 mm) was acquired along the direction of ACL with the axial FS-PDWI and coronal FS-PDWI as the scanning position map, which took 3 min and 24 s. The scanning method and the performance of ACL were shown in Fig. 1.

### Image analysis

All images were transmitted to Picture Archiving and Communication Systems (PACS) and independently reviewed by two musculoskeletal radiologists (the first reader with 8 years of experience and the second reader with 6 years of experience). They were blinded to clinical and arthroscopic results. The reading procedure started with standard MRI sequences and followed by combining the new modified DPP-TSO-Sag-FS-PDWI sequence into the standard sequences. The ACL injury on the MRI is divided into three grades according to the diagnostic criteria: grade I injury is a partial tear with less than half of ligament disrupted; grade II injury is a partial tear with more than half part of the ligament tear; grade III was a complete tear^16^.

### Arthroscopy protocol

The ACL reconstruction surgery was performed within one month following the MR examination, with the longest interval being 28 days and the shortest being the second day after the examination. The arthroscopy operation was performed by using American Smith & Nephew arthroscopic device. The procedure was performed by a senior surgeon with more than 10 years of experience in sports medicine and aware of MRI results and clinical reports. After successful anesthesia, the patient is placed in a supine position. Routine iodine disinfection, draping, membrane application, and blood drainage are performed. Under anesthesia, examinations such as the varus and valgus stress tests and anterior drawer test are conducted. Incisions are made on both sides of the knee joint gap, approximately 0.5 cm, to serve as the arthroscopic light source and instrument channel. The knee joint and its internal structures are examined. The ACL tears were classified into three levels according to the American Academy of Orthopedic Surgeons (AAOS) grading system: grade I is a sprain, mildly damaged and slightly stretched, but it can still keep the knee joint stable; grade II is a sprain and the ligament is stretched or loose, which is also known as partial tear; grade III is a sprain with complete tear, that is, the ligament breaks into two parts and the knee joint is unstable (https://orthoinfo.aaos.org/en/diseases--conditions/anterior-cruciate-ligament-acl-injuries/). All patients performed arthroscopic examination, but not all ACL injury patients underwent ACL reconstruction surgery. This decision was made by the senior surgeon based on surgical guidelines, individual patient characteristics, and the severity of the injury. Patients with normal ACLs, Grade I ACL injuries, and some Grade II ACL injuries (primarily small tears) did not undergo reconstruction surgery. ACL reconstruction surgery was performed on all Grade III injuries and some Grade II injuries (mainly extensive tears).

### Statistical analysis

The frequency of MRI diagnosis was counted, including standard MRI sequences and the new modified DPP-TSO-Sag-FS-PDWI sequences, compared to arthroscopy results. The χ^2^ test of paired comparison (Mc Nemer-Bowker) and weighted Kappa test was performed to compare the consistency of MRI diagnosis with arthroscopic results and the twice observations. When the kappa value ≤ 0.20, it indicates slight consistency; 0.21 to 0.40, fair consistency; 0.61 to 0.80, substantial consistency; 0.81 to 0.99, almost perfect consistency and 1.0, perfect consistency^17^. The sensitivity (SE), specificity (SP), accuracy (AC), positive predictive value (PPV), and negative predictive value (NPV) of MRI diagnosis were calculated for Grade I, Grade II, and Grade III based on true positive (TP), true negative (TN), false positive (FP), and false-negative (FN). The Statistic Package for Social Sciences (SPSS, version 25.0, Chicago, IL) software was used for statistical analysis, and* p* values < 0.05 were considered significant for 2-tailed probability.

### Ethics approval

This retrospective study was conducted according to the guidelines of the Declaration of Helsinki, and approved by the Institutional Ethics Committee of the First Affiliated Hospital of Soochow University (protocol code No.155, 13th of July 2020).

### Consent to participate

Informed consent was waived by the Institutional Ethics Committee of the First Affiliated Hospital of Soochow University because of the retrospective nature of the study and the use of anonymous clinical data.

## Results

### Patients’ characteristics

A total of 42 patients were analyzed in our retrospective study. Clinical data of the patients with the results of twice MRI observations and arthroscopy examination were shown in Table 1.Forty patients suffered an ACL injury and two patients suffered meniscus injury with normal ACL (suspected ACL injury before arthroscopy). There were 33 men with an average age (± standard deviation) of 29 ± 7 years and 9 women with 31 ± 12 years. According to the medical records, 73.8% (31/42) of them suffered from sports injuries and the rest 26.2% (21/42) patients suffered from accidents.

### MRI and arthroscopy manifestations in different grade of ACL injury

**Figure 2:**
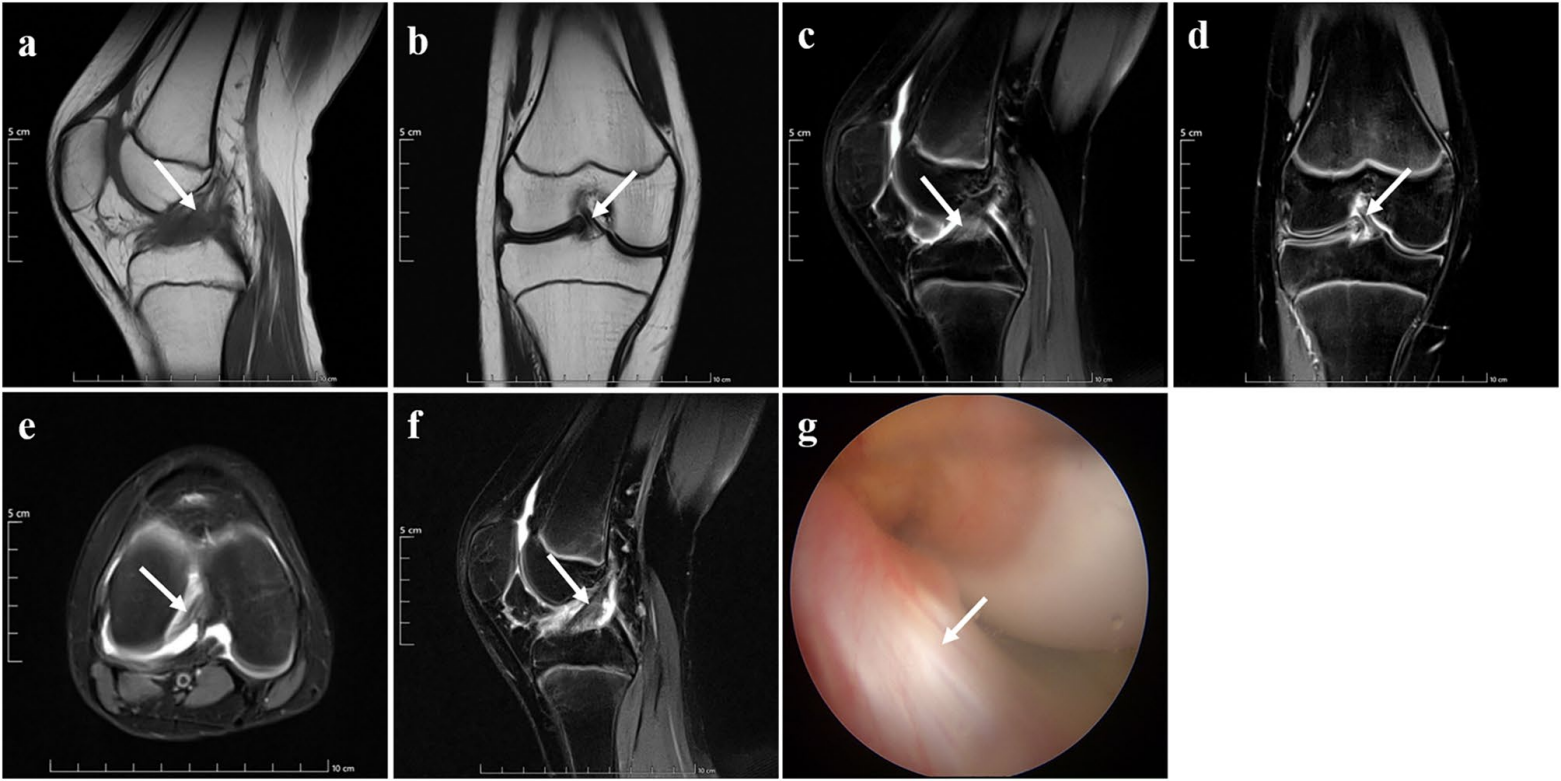
Normal ACL presence on MRI and arthroscopy of a 14-years-old female teenager. The ACL was shown on sagittal T_1_WI (**a**), coronal T_2_WI (**b**), Sagittal PDWI (**c**), coronal PDWI (**d**), and axial PDWI (**e**). It was tilted from the tibial to the condyle and the attachment was inserted from the condyle to the notch with low signal intensity (arrows). The ligament could be displayed completely on one level of the new modified DPP-TSO-Sag-FS-PDWI (arrow on (**f**)). The normal appearance of ACL was proved on arthroscopy (arrow on (**g**)).

Standard MRI sequences and the new modified DPP-TSO-Sag-FS-PDWI sequences showed similar results for the two normal ACLs, and the appearance of ligaments had normal signal intensity without any separation. Complete ligament shape and its low signal intensity could be visualized on one or continuous layers of the new modified DPP-TSO-Sag-FS-PDWI sequence. The arthroscopic finding revealed the normal ligament was tight and tilted from the tibia to the condyle in the oblique course (Fig. 2).

**Figure 3:**
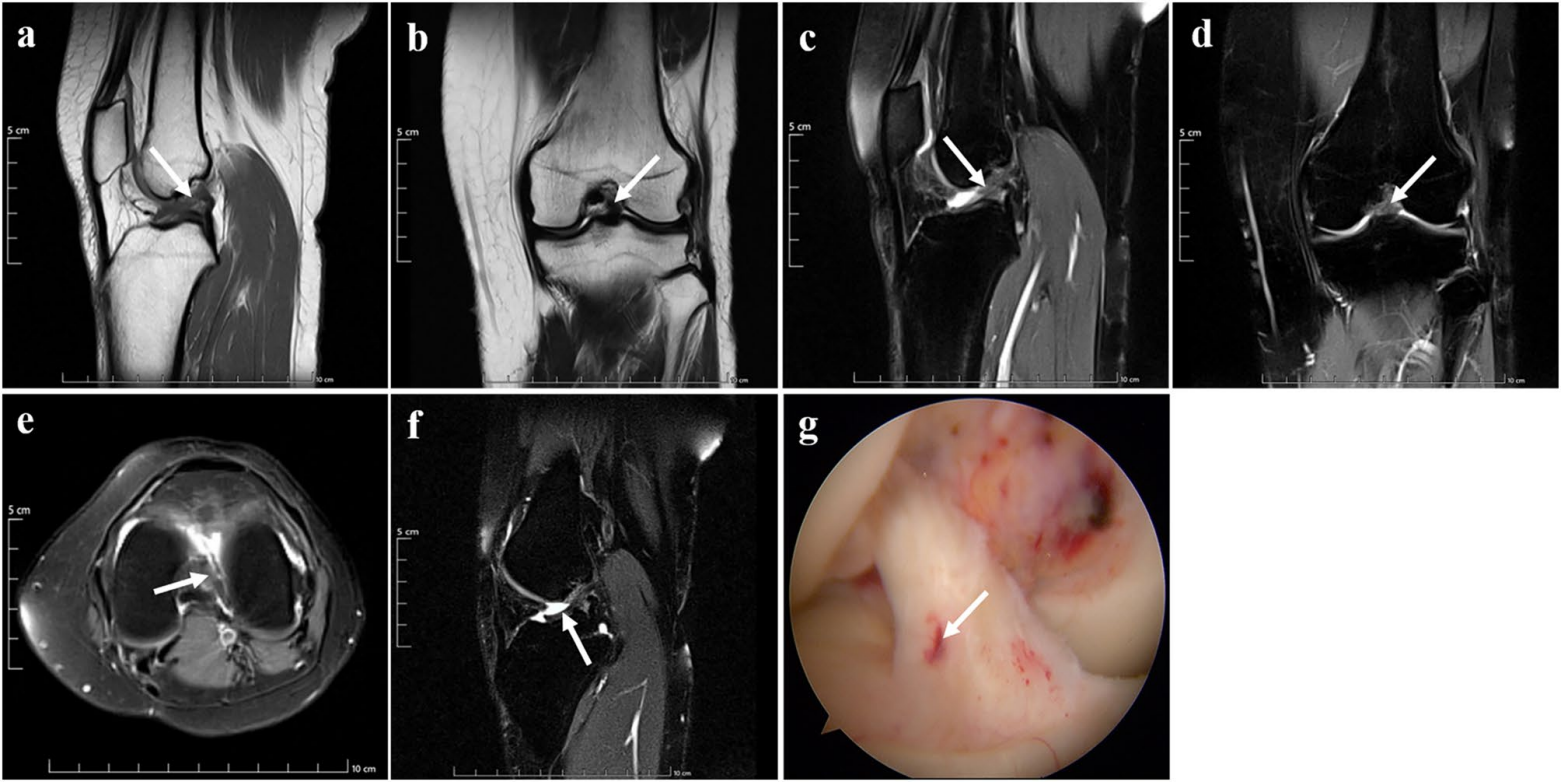
Grade I injury of ACL presence on MRI and arthroscopy of a 34-years-old woman. The shape and signal intensity of the ACL (arrows) was similar to the normal ligament on sagittal T_1_WI (**a**), coronal T_2_WI (**b**), Sagittal PDWI (**c**), coronal PDWI (**d**), and axial PDWI (**e**) with effusion of knee joint on PDWI. The ligament was tortuous as “*S*-shaped” and localized edema with increased signal intensity in the tibial part on the new modified DPP-TSO-Sag-FS-PDWI (arrow on (**f**)). Arthroscopy showed the ligament was loose with a hematoma in the anterior tibial part of the ligament (arrow on (**g**)).

Grade I injury of ACL is mild injury. The shape of the injured ligament was similar to the normal ligament on standard sequences. On the new modified DPP-TSO-Sag-FS-PDWI sequence, the ligament was tortuous with localized increased signal intensity. The arthroscopic examination revealed that the ligament was not torn, with minor localized bleeding, and a slight decrease in its elasticity (Fig. 3).

**Figure 4:**
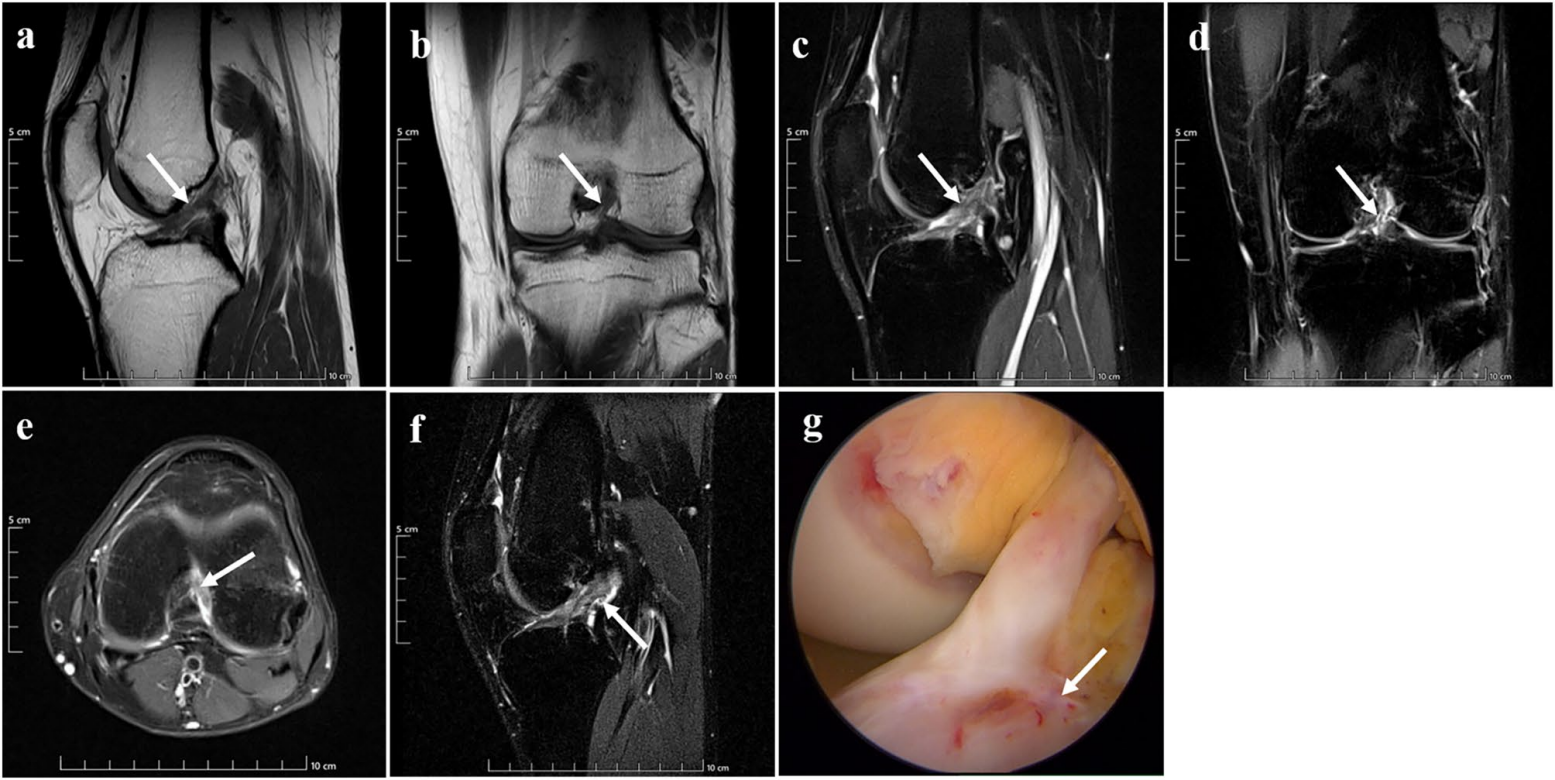
Grade II injury presence on MRI and arthroscopy of a 35-years-old man. The remaining structure of ACL was shown on sagittal and coronal T_1_WI (arrows on (**a** and **b**)). The ligament lost continuity and turned thinner, and its shape was distorted with a high signal on sagittal PDWI (arrow on (**c**)) and coronal PDWI (arrow on (**d**)). The signal of ACL increased and surrounded by the fluid on axial PDWI (arrow on (**e**)). It could be shown on the new modified DPP-TSO-Sag-FS-PDWI (**f**) that most of the ACL was swollen with the increased signal. A tear was seen at the posterior femoral part of the ACL, surrounded by fluid, and the free edge was reflexed (arrow), which was corresponded to the arthroscopic image. Arthroscopy showed that most remaining part of the ACL was still attached to the origin site and a small portion of the fibrous was tearing down in the middle of ACL (arrow on (**g**)).

The grade II injury of ACL showed partial tear with morphological change on standard sequences, it also manifested as local swelling, partial thinning of the ligament, and unclear tearing. Similar findings were also present on the new modified DPP-TSO-Sag-FS-PDWI sequence, but there was a separation in the part of the ligament. During the arthroscopic examination, grade II injury was characterized by partial tear with free edge and loose ligament stretch and tension (Fig. 4).

**Figure 5:**
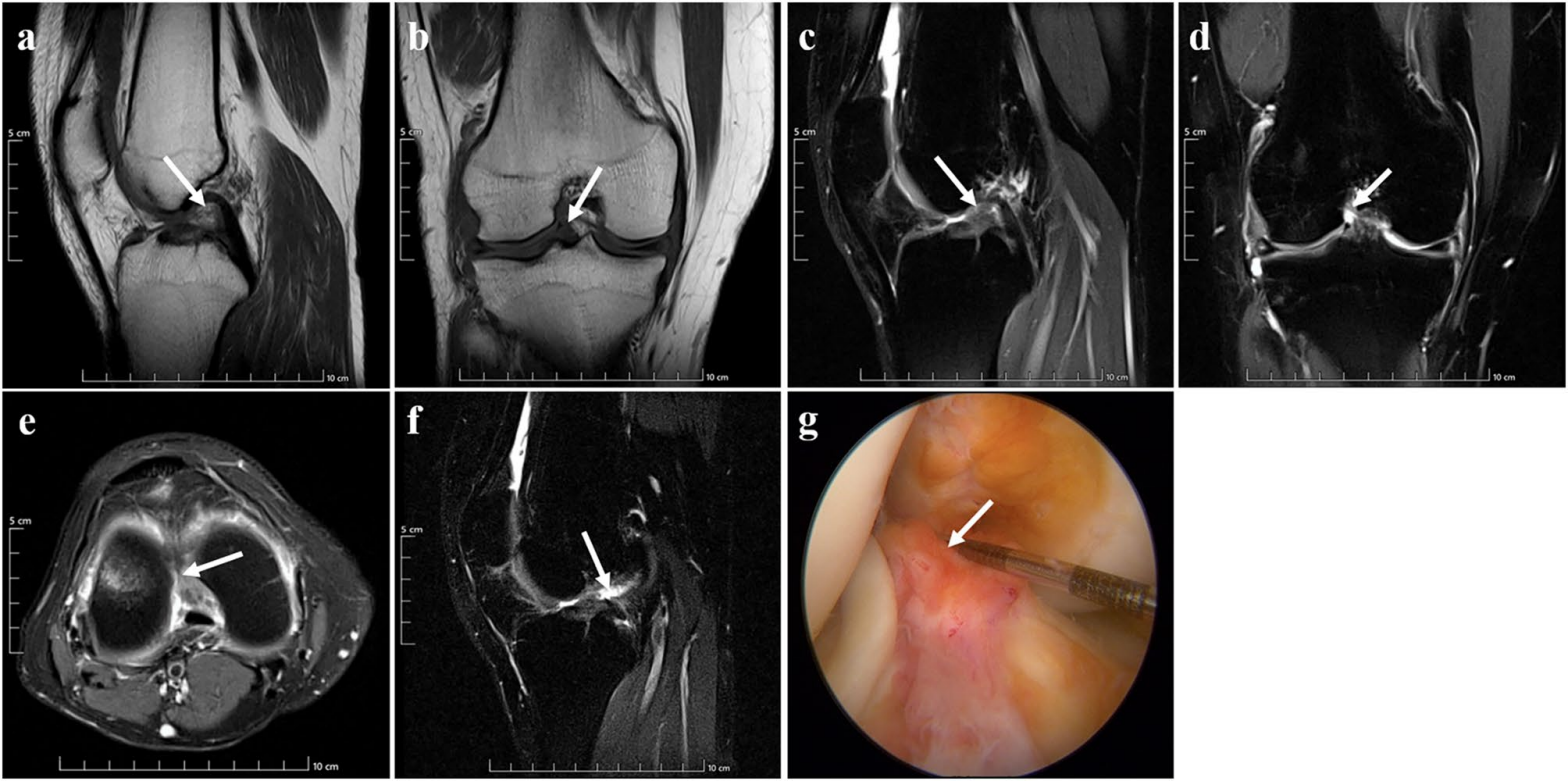
Grade III injury presence on MRI and arthroscopy of a 23-years-old woman. The absence of ligament integrity and continuity were shown on sagittal, and coronal T_1_WI (arrows on (**a** and **b**)). Remaining filamentous ACL with surrounding liquid (arrows) still could be seen on sagittal PDWI (**c**), coronal PDWI (**d**), and axial PDWI (**e**), which would cause misdiagnosis. The ligament was completely separated into two parts and the two damaged ends with their intermediate filled fluids (arrow) were displayed on the new modified DPP-TSO-Sag-FS-PDWI (**f**). Arthroscopy found the ligament was divided into two parts without continuity and completely separated, one of the damaged edges faced up (arrow on (**g**)).

The grade III injury of ACL on standard sequences and the new modified DPP-TSO-Sag-FS-PDWI sequence showed the absence of ligament integrity. The ligament was completely torn into two parts and the separation was obvious. Arthroscopic examination revealed a complete tear of the ligament (Fig. 5).

### Comparison of diagnostic consistency between MRI results and arthroscopy

We compared the diagnostic consistency of standard sequences and the new modified DPP-TSO-Sag-FS-PDWI sequence with arthroscopy for the first reader and second reader (Table 2). There was a fair consistency between standard sequences and arthroscopy in both reader 1 (*K* = 0.620, from 0.449 to 0.855) and reader 2 (*K* = 0.683, from 0.436 to 0.844). The consistency between the new modified DPP-TSO-Sag-FS-PDWI sequence and arthroscopy were almost perfect (reader 1: *K* = 0.876, from 0.707 to 1; reader 2: *K* = 0.833, from 0.696 to 0.996).

**Table 2:** The diagnostic consistency comparison of standard MRI sequences and the new modified DPP-TSO-SAG-FS-PDWI sequence with arthroscopy, and standard sequences with the new modified DPP-TSO-SAG-FS-PDWI sequence for reader 1 and reader 2.

Diagnostic consistency comparison		Standard sequences 1 and arthroscopy		Standard sequences 2 and arthroscopy		DPP-TSO-Sag-FS-PDWI 1 and arthroscopy		DPP-TSO-Sag-FS-PDWI 2 and arthroscopy	
Standard sequences and DPP-TSO-Sag-FS-PDWI comparied with arthroscopy	McNemar-Bowker test	*χ*^2^ = 5.571	*p* = 0.134	*χ*^2^ = 3.200	*p* = 0.362	*χ*^2^ = 0.333	*p* = 0.564	*χ*^2^ = 0.000	*p* = 1.000
	Weighted Kappa	*K* = 0.620 (0.449 to 0.855)	*p* = 0.000	*K* = 0.683 (0.436 to 0.844)	*p* = 0.000	*K* = 0.876 (0.707 to 1)	*p* = 0.000	*K* = 0.833 (0.696 to 0.996)	*p* = 0.000

Diagnostic consistency comparison		Standard sequences 1 and DPP-TSO-Sag-FS-PDWI 1		Standard sequences 2 and DPP-TSO-Sag-FS-PDWI 2		Standard sequences 1 and standard sequences 2		DPP-TSO-Sag-FS-PDWI 1 and DPP-TSO-Sag-FS-PDWI 2	
Standard sequences comparied with DPP-TSO-Sag-FS-PDWI sequence	McNemar-Bowker test	*χ*^2^ = 7.000	*p* = 0.072	*χ*^2^ = 4.000	*p* = 0.261	*χ*^2^ = 4.571	*p* = 0.206	*χ*^2^ = 0.200	*p* = 0.655
	Weighted Kappa	*K* = 0.710 (0.544 to 0.916)	*p* = 0.000	*K* = 0.722 (0.512 to 0.880)	*p* = 0.000	*K* = 0.598 (0.338 to 0.805)	*p* = 0.000	*K* = 0.794 (0.537 to 0.923)	*p* = 0.000

DPP-TSO-Sag-FS-PDWI, Dual precision positioning of thin-slice oblique sagittal fat suppression proton density weighted imaging; 1, Reader 1; 2, Reader 2.

The diagnostic consistency comparison between standard sequences and the new modified DPP-TSO-Sag-FS-PDWI sequence for both readers were presented in Table 2. There was a fair consistency between them in both reader 1 (*K* = 0.710, from 0.544 to 0.916) and reader 2 (*K* = 0.722, from 0.512 to 0.880). Nevertheless, the diagnostic consistency of standard sequences was only 0.598 (0.338 to 0.805) and the new modified DPP-TSO-Sag-FS-PDWI sequence was 0.794 (0.537 to 0.923), thus indicating good diagnostic repeatability of the new modified DPP-TSO-Sag-FS-PDWI sequence.

### Diagnostic accuracy of standard MRI and DPP-TSO-Sag-FS-PDWI sequences compared to arthroscopy

The number of MRI diagnostic results for the different grades of tears in each sequence was calculated, including TP, FN, FP, and TN (Supplementary Material), which were compared to arthroscopic examination results.

The diagnostic effectiveness of standard sequences and the new modified DPP-TSO-Sag-FS-PDWI sequence for the reader 1 was presented in Table 3, including sensitivity, specificity, accuracy, positive predictive values (PPV), and negative predictive values (NPV). Compared to standard sequences, the new modified DPP-TSO-Sag-FS-PDWI sequence showed superior sensitivity in the diagnosis of grade I (100% vs. 75%) and grade III injury (95.7% vs. 77.8%), better specificity for both grade II (96.4% vs. 82.4%), higher accuracy for grade III (92.9% vs. 78.6%) superior PPV for grade II (92.3% vs. 53.9%) and greater NPV for grade III (94.4% vs. 66.7%). Both sequences showed similar sensitivity for grade II injury (85.7% vs. 87.5%), similar specificity for grade III (89.5% vs. 80%), similar accuracy for grade I (100% vs. 97.6%) and grade II (92.9% vs. 83.3%). Furthermore, the specificity and PPV values of both sequences were the same for grade I injury (100%).

**Table 3:** The SE, SP, AC, PPV and NPV percentage values of three grades of ACL tears on standard MRI and the new modified DPP-TSO-Sag-FS-PDWI Sequences for reader 1 and reader 2.

Grade		I	II	III
SE (%)	Standard sequences 1	75	87.5	77.8
	DPP-TSO-Sag-FS-PDWI 1	100	85.7	95.7
	Standard sequences 2	60	75	90.9
	DPP-TSO-Sag-FS-PDWI 2	100	84.6	91.7
SP (%)	Standard sequences 1	100	82.4	80
	DPP-TSO-Sag-FS-PDWI 1	100	96.4	89.5
	Standard sequences 2	100	86.7	80
	DPP-TSO-Sag-FS-PDWI 2	100	93.1	88.9
AC (%)	Standard sequences 1	97.6	83.3	78.6
	DPP-TSO-Sag-FS-PDWI 1	100	92.9	92.9
	Standard sequences 2	95.2	83.3	85.7
	DPP-TSO-Sag-FS-PDWI 2	100	90.5	90.5
PPV (%)	Standard sequences 1	100	53.9	87.5
	DPP-TSO-Sag-FS-PDWI 1	100	92.3	91.7
	Standard sequences 2	100	69.2	83.3
	DPP-TSO-Sag-FS-PDWI 2	100	84.6	91.7
NPV (%)	Standard sequences 1	97.4	96.6	66.7
	DPP-TSO-Sag-FS-PDWI 1	100	93.1	94.4
	Standard sequences 2	94.9	89.7	88.9
	DPP-TSO-Sag-FS-PDWI 2	100	93.1	88.9

DPP-TSO-Sag-FS-PDWI, Dual precision positioning of thin-slice oblique sagittal fat suppression proton density weighted imaging; 1, Reader 1; 2, Reader 2, *SE* sensitivity, *SP* specificity, *AC* accuracy, *PPV* positive predictive value, *NPV* negative predictive value.

The diagnostic indicators of the reader 2 of standard sequences and the new modified DPP-TSO-Sag-FS-PDWI sequence were presented in Table 3. Compared to standard sequences, the new modified DPP-TSO-Sag-FS-PDWI sequence showed greater sensitivity for grade I (100% vs. 60%) and grade II injury (84.6% vs. 75%), higher specificity for grade II (93.1% vs. 86.7%) and grade III (88.9% vs. 80%), better accuracy for all grades [(100% vs. 95.2%), (90.5% vs. 83.3%) and (90.5% vs. 85.7%)] and superior PPV for grade II (84.6% vs. 69.2%). Furthermore, both sequences showed similar sensitivity for grade III (91.7% vs. 90.9%), same specificity and PPV for grade I (100%), and same NPV for grade III (88.9%).

## Discussion

The misdiagnosis rate of anterior cruciate ligament (ACL) tear of the knee joint is as high as 47% due to the confusion of high signal generated by synovial hyperplasia and partial volume effect on the standard MRI sequences^8^. A study revealed the evaluation of ACL tear by standard MRI sequences with additional oblique-sagittal plane showed high sensitivity and specificity^15,18^. Furthermore, another study using two additional oblique sequences (sagittal and coronal) to evaluate the ACL tear concluded that the use of oblique coronal and oblique sagittal MRI of the knee improved the diagnostic accuracy of the ACL tear^19^. Therefore, we performed this study to analyze the diagnostic accuracy by adding a new modified dual precision positioning of thin-slice oblique sagittal fat suppression proton-density weighted imaging (DPP-TSO-Sag-FS-PDWI) to standard MRI sequences. The diagnostic consistency of the new modified DPP-TSO-Sag-FS-PDWI sequence and arthroscopy was better than standard MRI sequences for both reader 1 (*K* = 0.876 vs. 0.620) and reader 2 (*K* = 0.833 vs. 0.683) with good diagnostic repeatability (*K* = 0.794 vs. 0.598). Compared to standard sequences, the new modified DPP-TSO-Sag-FS-PDWI sequence can accurately classify and correctly diagnose the grades of ACL injury, whose sensitivity, specificity, accuracy, positive predictive value (PPV), and negative predictive value (NPV) were all greater than 84%, especially for grade II injury (partial tear) as the PPV value was superior for the reader 1 (92.3% vs. 53.9%) and reader 2 (84.6% vs. 69.2%).

Previous studies have used standard MRI sequences with or without the additional sequence to diagnose an ACL tear. A previous study using 3.0-T MRI with standard sequences revealed that the specificity and accuracy of the partial injury (97%, 95%) and complete injury (99%, 97%), which are similar to present study. However, the sensitivity of the partial injury (77%) was lower than our study (85.7% or 84.6%)^20^. This indicates that although 3.0-T MRI is a highly accurate method for the diagnosis of ACL injuries, the new modified DPP-TSO-Sag-FS-PDWI sequence on a 1.5-T scanner with a single channel knee coil can achieve similar effects and further improve the diagnostic sensitivity to partial tears. There was a study of 149 patients with three different methods, including standard MRI sequences, patient knee in 17-degree flexion MRI technique, and additional sagittal-oblique sequence, and it revealed the sagittal-oblique sequence more sensitive (*p* < 0.001) than standard MRI sequences. The author mentioned the oblique-sagittal technique can display the partial tear because of the double angulation which is following the course of the patient’s ligament due to the approximate orientation of the external rotation of the foot^15^. The study using two additional oblique sequences (sagittal and coronal) to evaluate the ACL tear concluded that the usage of oblique coronal and oblique sagittal MRI of the knee improved the diagnostic accuracy of the ACL tear^19^. Furthermore, another study revealed the evaluation of ACL tear by standard MRI sequences with additional oblique-sagittal plane showed high sensitivity and specificity compared to a standard MRI protocol^18^. The results in these studies are similar to ours and we further found that our new modified DPP-TSO-Sag-FS-PDWI sequence can maximize the acquisition of ACL complete and true anatomical images and exclude the influence of anatomical differences between individuals, which is helpful to improve the authenticity and accuracy of ACL scanning images and to improve the diagnostic accuracy of ACL injury with its three grades.

To our knowledge, this is the first study to display and diagnose the ACL injury with its three grades by using a dual precision positioning based on axial and coronal localization. Our study not only validated the diagnostic accuracy of standard sequences used to diagnose ACL injury and its grades but also added a new modified dual precision positioning of thin-slice oblique sagittal sequence to improve the diagnostic accuracy. By furnishing more precise diagnostic outcomes, our approach holds the potential to reduce the necessity for exploratory arthroscopic surgeries, particularly when MRI unequivocally indicates the absence of ACL injury. This, in turn, can lead to a more cost-effective and patient-friendly diagnostic approach^9^. Additionally, our scanning method is simple, the scanning time is moderate, and there is no special requirement for the MR scanner and the patient's position. Therefore, it is very suitable for popularization and application in clinical work.

While our study has yielded some valuable insights, it is essential to acknowledge several significant limitations. The primary limitations include a small sample size, constraints related to the control group, and a lack of statistical considerations. Firstly, we acknowledge that our study only included a very small number of patients who did not experience ACL tears. This, to some extent, restricts our in-depth exploration of normal ACL conditions and grade I injuries. The reason behind this limitation is that patients with normal ACLs, grade I injuries, and some grade II ACL injuries (mainly minor tears) often do not require ACL reconstruction surgery. Consequently, obtaining arthroscopic examination results for these patients proved challenging. This limitation resulted in a small number of patients in our normal control group, particularly concerning grade I injuries. Secondly, we did not conduct a formal sample size estimation, implying that our study may suffer from statistical inadequacies. This issue primarily stems from the restricted patient recruitment and our inability to acquire an adequate number of patients for the control group. This has led to a relatively small sample size, limiting the generalizability of certain observations. The imbalance in sample distribution has biased our study towards comparing different degrees of ACL injuries rather than comparing to a control group with intact ACLs. Consequently, it restricts a comprehensive understanding of ACL integrity and may not apply to a broader spectrum of ACL injury cases. Additionally, we cannot rule out potential biases such as selection bias and information bias.

In the future, our research direction includes increasing the sample size to enhance statistical power, especially by expanding the control group. We plan to achieve this through broader multicenter collaborations or long-term longitudinal studies. Moreover, we are collaborating with expert clinical statisticians to consider more robust statistical methods to obtain more reliable results within the constraints of a limited sample size. Finally, our planned further research may focus on assessing the radiographic features of various types of ACL injuries, including those unrelated to ACL tears, to gain a better understanding of the diversity of ACL injuries.

## Conclusions

The new modified DPP-TSO-Sag-FS-PDWI sequence can clearly display the ligament injury on one or continuous levels by maximizing the acquisition of ACL complete and true anatomical images and excluding the influence of anatomical differences between individuals. It can improve the diagnostic accuracy with good diagnostic repeatability and classify three different grades of ACL injury accurately, especially for grade II injury. This new modified sequence also assists the physicians to reduce unnecessary arthroscopic intervention and provides a precise diagnostic and treatment basis for ACL injury.

### Supplementary Information

Supplementary Information 1.

Supplementary Information 2.

## Data Availability

The datasets generated and/or analysed during the current study are available in the Google Drive. The link is https://drive.google.com/file/d/1FGOTOTJ6vi6K494Nnh947hBP7YrfQ4kj/view?usp=sharing. The datasets analyzed during this study are available from the corresponding author upon reasonable request.
