# Supplementary material for: A new modified MR dual precision positioning of thin-slice oblique sagittal fat suppression proton density weighted imaging: its diagnostic accuracy in anterior cruciate ligament injury
Source: Sci Rep. 2024 Jan 3;13:23109. doi: 10.1038/s41598-023-50909-4 (PMC10764300; doi:10.1038/s41598-023-50909-4)
Supplement: Supplementary file 1 — Supplementary Information 1. [file 41598_2023_50909_MOESM1_ESM.docx]

Distribution of TP, FN, FP and TN of the three grade of ACL injury on MRI

| MRI sequences | Grade I | | | | Grade II | | | | Grade III | | | |
| --- | --- | --- | --- | --- | --- | --- | --- | --- | --- | --- | --- | --- |
|  | TP | FN | FP | TN | TP | FN | FP | TN | TP | FN | FP | TN |
| Standard sequences 1 | 3 | 0 | 1 | 38 | 7 | 6 | 1 | 28 | 21 | 3 | 6 | 12 |
| DPP-TSO-Sag-FS-PDWI 1 | 3 | 0 | 0 | 39 | 12 | 1 | 2 | 27 | 22 | 2 | 1 | 17 |
| Standard sequences 2 | 3 | 0 | 2 | 37 | 9 | 4 | 3 | 26 | 20 | 4 | 2 | 16 |
| DPP-TSO-Sag-FS-PDWI 2 | 3 | 0 | 0 | 39 | 11 | 2 | 2 | 27 | 22 | 2 | 2 | 16 |

DPP-TSO-Sag-FS-PDWI, Dual precision positioning of thin-slice oblique sagittal fat suppression proton density weighted imaging; 1, Reader 1; 2, Reader 2; TP, True positive; FN, False negative; FP, False positive; and TN, True negative.
