# Supplementary figures and images for: A new modified MR dual precision positioning of thin-slice oblique sagittal fat suppression proton density weighted imaging: its diagnostic accuracy in anterior cruciate ligament injury
Source: Sci Rep. 2024 Jan 3;13:23109. doi: 10.1038/s41598-023-50909-4 (PMC10764300; doi:10.1038/s41598-023-50909-4)

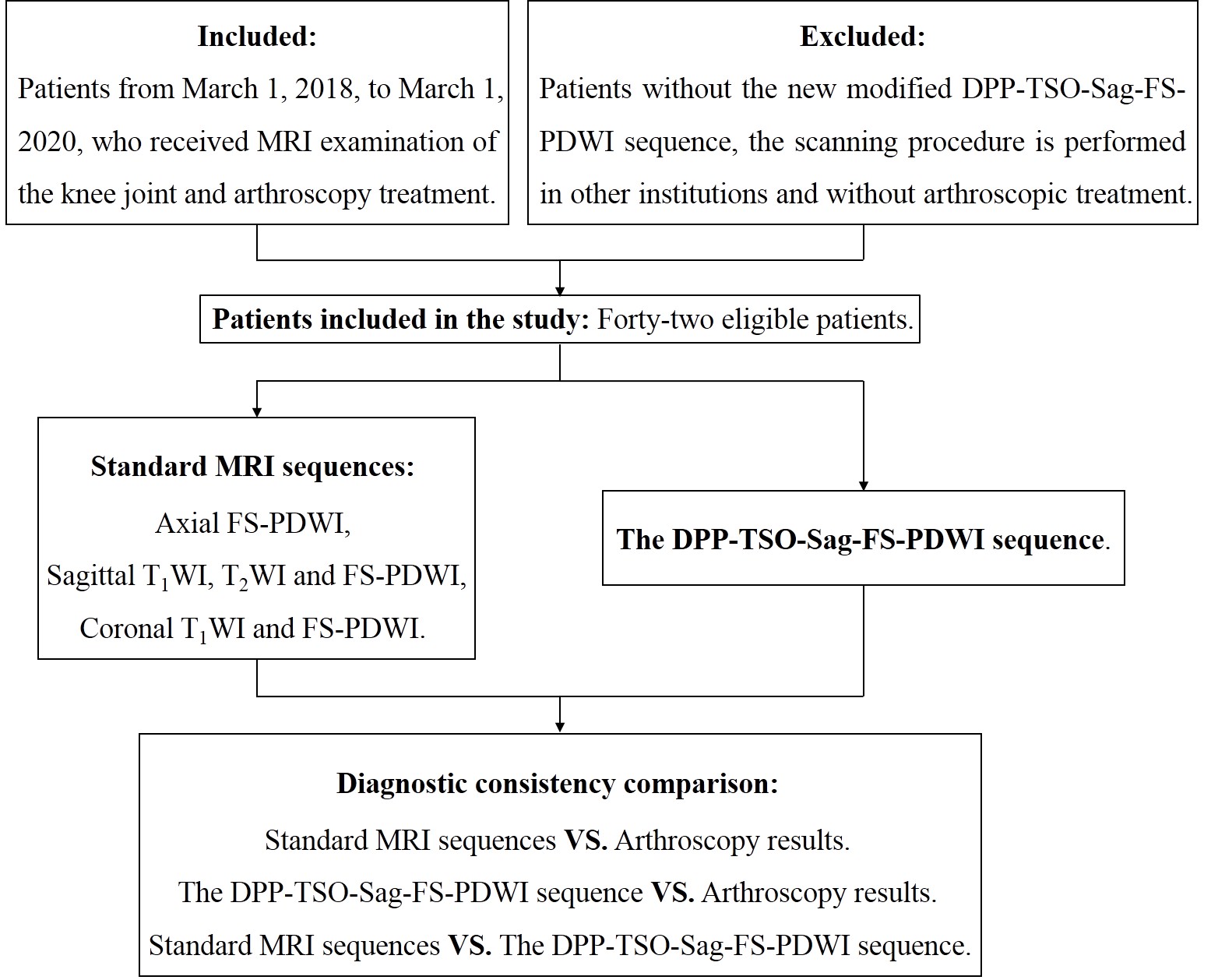

Supplement: Supplementary file 2 — Supplementary Information 2. [file 41598_2023_50909_MOESM2_ESM.jpg]
